# The Database of Cross-Linguistic Colexifications, reproducible analysis of cross-linguistic polysemies

**DOI:** 10.1038/s41597-019-0341-x

**Published:** 2020-01-13

**Authors:** Christoph Rzymski, Tiago Tresoldi, Simon J. Greenhill, Mei-Shin Wu, Nathanael E. Schweikhard, Maria Koptjevskaja-Tamm, Volker Gast, Timotheus A. Bodt, Abbie Hantgan, Gereon A. Kaiping, Sophie Chang, Yunfan Lai, Natalia Morozova, Heini Arjava, Nataliia Hübler, Ezequiel Koile, Steve Pepper, Mariann Proos, Briana Van Epps, Ingrid Blanco, Carolin Hundt, Sergei Monakhov, Kristina Pianykh, Sallona Ramesh, Russell D. Gray, Robert Forkel, Johann-Mattis List

**Affiliations:** 1 Department of Linguistic and Cultural Evolution, Max Planck Institute for the Science of Human History, Jena, Germany; 2 ARC Centre of Excellence for the Dynamics of Language, Australian National University, Canberra, Australia; 3 Stockholm University, Stockholm, Sweden; 4 Friedrich Schiller University, Jena, Germany; 5 SOAS, London, UK; 6 CNRS LLACAN, Paris, France; 7 University of Leiden, Leiden, Netherlands; 8 Independent English-Chinese Translator and linguistic researcher, Taipei, Taiwan; 9 University of Helsinki, Helsinki, Finland; 10 University of Oslo, Oslo, Norway; 11 University of Tartu, Tartu, Estonia; 12 Lund University, Lund, Sweden

**Keywords:** Databases, Sociology

## Abstract

Advances in computer-assisted linguistic research have been greatly influential in reshaping linguistic research. With the increasing availability of interconnected datasets created and curated by researchers, more and more interwoven questions can now be investigated. Such advances, however, are bringing high requirements in terms of rigorousness for preparing and curating datasets. Here we present CLICS, a Database of Cross-Linguistic Colexifications (CLICS). CLICS tackles interconnected interdisciplinary research questions about the colexification of words across semantic categories in the world’s languages, and show-cases best practices for preparing data for cross-linguistic research. This is done by addressing shortcomings of an earlier version of the database, CLICS2, and by supplying an updated version with CLICS3, which massively increases the size and scope of the project. We provide tools and guidelines for this purpose and discuss insights resulting from organizing student tasks for database updates.

## Abstract (summary)

**Table:** 

Measurement(s)	Meaning • word
Technology Type(s)	digital curation • computational modeling technique • Semantics
Factor Type(s)	source data • language

Machine-accessible metadata file describing the reported data: 10.6084/m9.figshare.11317625

## Background & Summary

The quantitative turn in historical linguistics and linguistic typology has dramatically changed how scholars create, use, and share linguistic information. Along with the growing amount of digitally available data for the world’s languages, we find a substantial increase in the application of new quantitative techniques. While most of the new methods are inspired by neighboring disciplines and general-purpose frameworks, such as evolutionary biology^1,2^, machine learning^3,4^, or statistical modeling^5,6^, the particularities of cross-linguistic data often necessitate a specific treatment of materials (reflected in recent standardization efforts^7,8^) and methods (illustrated by the development of new algorithms tackling specifically linguistic problems^9,10^).

The increased application of quantitative approaches in linguistics becomes particularly clear in *semantically oriented* studies on *lexical typology*, which investigate how languages distribute meanings across their vocabularies. Although questions concerning such categorizations across human languages have a long-standing tradition in linguistics and philosophy^11,12^, global-scale studies have long been restricted to certain recurrent semantic fields, such as *color terms*^13,14^, *kinship terms*^15,16^, and *numeral systems*^17^, involving smaller amounts of data with lower coverage of linguistic diversity in terms of families and geographic areas.

Along with improved techniques in data creation and curation, advanced computational methods have opened new possibilities for research in this area. One example is the *Database of Cross-Linguistic Colexifications*, first published in 2014^18^, which offers a framework for the computer-assisted collection, computation, and exploration of worldwide patterns of cross-linguistic “colexifications”. The term *colexification*^19^ refers to instances where the same word expresses two or more comparable concepts^20,21^, such as in the common case of *wood* and *tree* “colexifying” in languages like Russian (both expressed by the word *dérevo*) or Nahuatl (both *k*^*w*^ *awi-t*). By harvesting colexifications across multiple languages, with recurring patterns potentially reflecting universal aspects of human perception and cognition, researchers can identify cross-linguistic polysemies without resorting to intuitive decisions about the motivation for such identities.

**Fig. 1:**
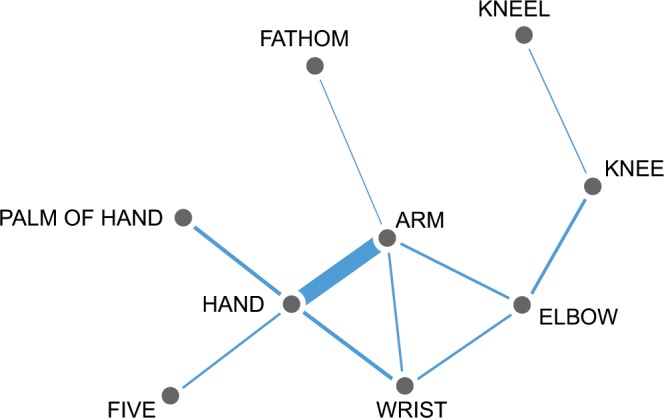
Example of a colexification network. A strong link between ARM and HAND is shown, showing that in many languages both concepts are expressed with the same word; among others, weaker links between concepts HAND and FIVE, explainable by the number of fingers on a hand, and ELBOW and KNEE, explainable as both being joints, can also be observed.

The CLICS project reflects the rigorous and transparent approaches to standardization and aggregation of linguistic data, allowing to investigate colexifications through global and areal semantic networks, as in the example of Fig. 1, mostly by reusing data first collected for historical linguistics. We designed its framework, along with the corresponding interfaces, to facilitate the exploration and testing of alleged cross-linguistic polysemies^22^ and areal patterns^23–25^. The project is becoming a popular tool not only for examining cross-linguistic patterns, particularly those involving unrelated languages, but also for conducting new research in fields not strictly related to semantically oriented lexical typology^26–30^ in its relation to semantic typology^31–33^.

A second version of the CLICS database was published in 2018, revising and greatly increasing the amount of cross-linguistic data^34^. These improvements were made possible by an enhanced strategy of *data aggregation*, relying on the standardization efforts of the Cross-Linguistic Data Formats initiative (CLDF)^7^, which provides standards, tools, and best practice examples for promoting linguistic data which is FAIR: *findable*, *accessible*, *interoperable*, and *reusable*^35^. By adopting these principles and coding independently published cross-linguistic datasets according to the specifications recommended by the CLDF initiative, it was possible to increase the amount of languages from less than 300 to over 2000, while expanding the number of concepts in the study from 1200 to almost 3000.

A specific shortcoming of this second release of CLICS was that, despite being based on CLDF format specifications, it did not specify how data conforming to such standards could be created in the first place. Thus, while the CLDF data underlying CLICS2 are findable, accessible, interoperable, and reusable, the procedures involving their creation and expansion were not necessarily easy to apply due to a lack of transparency.

In order to tackle this problem, we have developed guidelines and software tools that help converting existing linguistic datasets into the CLDF format. We tested the suitability of our new curation framework by conducting two student tasks in which students with a background in linguistics helped us to convert and integrate data from different sources into our database. We illustrate the efficiency of this workflow by providing an updated version of our data, which increases the number of languages from 1220 to 3156 and the number of concepts from 2487 to 2906. In addition, we also increased and enhanced the transparency, flexibility, and reproducibility of the workflow by which CLDF datasets are analyzed and published within the CLICS framework, by publishing a testable virtual container^36^ that can be freely used on-line in the form of a *Code Ocean capsule*^37^.

## Methods

### Create and curate data in CLDF

The CLDF initiative promotes principles, tools, and workflows to make data cross-linguistically compatible and comparable, facilitating interoperability without strictly enforcing it or requiring linguists to abandon their long-standing data management conventions and expectations. Key aspects of the data format advanced by the initiative are an exhaustive and principled use of reference catalogs, such as Glottolog^38^ for languages and Concepticon^39^ for comparative concepts, along with standardization efforts like the Cross-Linguistic Transcription Systems (CLTS) for normalizing phonological transcriptions^8,40^.

**Fig. 2:**
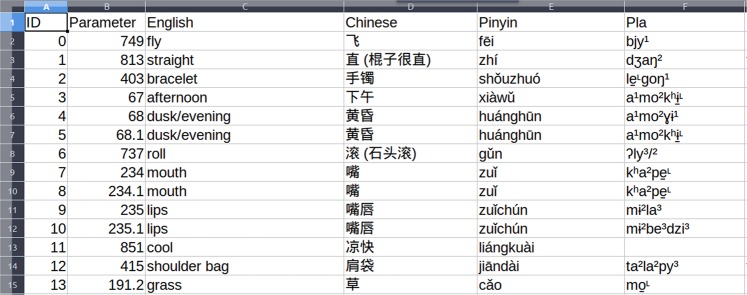
Raw data as a starting point for applying the data curation workflow. The table shows a screenshot of a snippet from the source of the yanglalo dataset.

Preparing data for CLICS starts with obtaining and expanding raw data, often in the form of Excel tables (or similar formats) as shown in Fig. 2.

By using our sets of tools, data can be enriched, cleaned, improved, and made ready for usage in multiple different applications, both current ones, such as CLICS, or future ones, using compliant data.

**Fig. 3:**
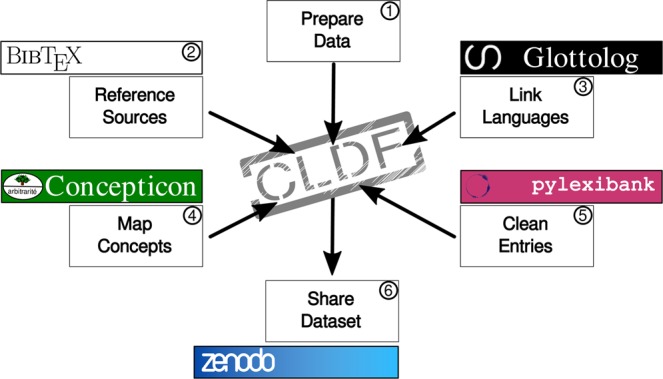
A diagram representing the six fundamental steps of a CLDF dataset preparation workflow.

This toolbox of components supports the creation and release of CLDF datasets through an integrated workflow comprising six fundamental steps (as illustrated in Fig. 3). First, (1) scripts prepare raw data from sources for digital processing, leading the way to the subsequent catalog cross-referencing at the core of CLDF. This task includes the steps of (2) referencing sources in the BibTeX format, (3) linking languages to Glottolog, and (4) mapping concepts to Concepticon. To guarantee straightforward processing of lexical entries by CLICS and other systems, the workflow might also include a step for (5) cleaning lexical entries of systematic errors and artifacts from data conversion. Once the data have been curated and the scripts for workflow reproducibility are completed, the dataset is ready for (6) public release as a package relying on the pylexibank library, a step that includes publishing the CLDF data on Zenodo and obtaining a DOI.

The first step in this workflow, preparing source data for digital processing (1), varies according to the characteristics of each dataset. The procedure ranges from the digitization of data collections only available as book scans or even fieldwork notes (using software for optical character recognition or manual labor, as done for the beidasinitic dataset^41^ derived from^42^), via the re-arrangement of data distributed in word processing or spreadsheet formats such as docx and xlsx (as for the castrosui dataset^43^, derived from^44^), up to extracting data from websites (as done for diacl^45^, derived from^46^). In many cases, scholars helped us by sharing fieldwork data (such as yanglalo^47^, derived from^48^, and bodtkhobwa^49^, derived from^50^), or providing the unpublished data underlying a previous publication (e.g. satterthwaitetb^51^, derived from^52^). In other cases, we profited from the digitization efforts of large documentation projects such as STEDT^53^ (the source of the suntb^54^ dataset, originally derived from^55^), and Northeuralex^56,57^.

In the second step, we identify all relevant sources used to create a specific dataset and store them in BibTeX format, the standard for bibliographic entries required by CLDF (2). We do this on a per-entry level, guaranteeing that for each data point it will always be possible to identify the original source; the pylexibank library will dutifully list all rows missing bibliographic references, treating them as incomplete entries. Given the large amount of bibliographic entries from language resources provided by aggregators like Glottolog^38^, this step is usually straightforward, although it may require more effort when the original dataset does not properly reference its sources.

The third and fourth steps comprise linking language varieties and concepts used in a dataset to the Glottolog (3) and the Concepticon catalogs (4), respectively. Both such references are curated on publicly accessible GitHub repositories, allowing researchers easy access to the entire catalog, and enabling them to request changes and additions. In both cases, on-line interfaces are available for open consultation. While these linking tasks require some linguistic expertise, such as for distinguishing the language varieties involved in a study, both projects provide libraries and tools for semi-automatic mapping that facilitate and speed up the tasks. For example, the mapping of concepts was tedious in the past when the entries in the published concept lists differed too much from proper glosses, such as when part-of-speech information was included along with the actual meaning or translation, often requiring a meticulous comparison between the published work and the corresponding concept lists. However, the second version of Concepticon^58^ introduced new methods for semi-automatic concept mapping through the pyconcepticon package, which can be invoked from the command-line, as well as a lookup-tool allowing to search concepts by fuzzy matching of elicitation glosses. Depending on the size of a concept list, this step can still take several hours, but the lookup procedure has been improved in the last version, because of the increasing number of concepts and concept lists.

In a fifth step, we use the pylexibank API to clean and standardize lexical entries, and remove systematic errors (5). This API allows users to convert data in raw format – when bibliographic references, links to Glottolog, and mappings to Concepticon are provided – to proper CLDF datasets. Given that linguistic datasets are often inconsistent regarding lexical form rendering, the programming interface is used to automatically clean the entries by (a) splitting multiple synonyms from their original *value* into unique *forms* each, (b) deleting brackets, comments, and other parts of the entry which do not reflect the original word form, but authors’ and compilers’ comments, (c) making a list of entries to ignore or correct, in case the automatic routine does not capture all idiosyncrasies, and (d) using explicit mapping procedures for converting from orthographies to phonological transcriptions. The resulting CLDF dataset contains both the original and unchanged textual information, labeled *Value*, and its processed version, labeled *Form*, explicitly informing what is taken from the original source and what results from our manipulations, always allowing to compare the original and curated state of the data. Even when the original is clearly erroneous, for example due to misspellings, the *Value* is left unchanged and we only correct the information in the *Form*.

As a final step, CLDF datasets are publicly released (6). The datasets live as individual Git repositories on GitHub that can be anonymously accessed and cloned. A dataset package contains all the code and data resources required to recreate the CLDF data locally, as well as interfaces for easily installing and accessing the data in any Python environment. Packages can be frozen and released on platforms like Zenodo, supplying them with persistent identifiers and archiving for reuse and data provenance. The datasets for CLICS3, for example, are aggregated within the CLICS Zenodo community (https://zenodo.org/communities/clics/, accessed on November 15, 2019).

Besides the transparency in line with the best practices for open access and reproducible research, the improvements to the CLICS project show the efficiency of this workflow and of the underlying initiative. The first version^18^ was based on only four datasets publicly available at the time of its development. The project was well received and reviewed, particularly due to the release of its aggregated data in an open and reusable format, but as a cross-linguistic project it suffered from several shortcomings in terms of data *coverage*, being heavily biased towards European and South-East Asian languages. The second version of CLICS^34^ combined 15 different datasets already in CLDF format, making data reuse much easier, while also increasing quality and coverage of the data. The new version doubles the number of datasets without particular needs for changes in CLICS itself. The project is fully integrated with Lexibank and with the CLDF libraries, and, as a result, when a new dataset is published, it can be installed to any local CLICS setup which, if instructed to rebuild its database, will incorporate the new information in all future analyses. Likewise, it is easy to restrict experiments by loading only a selected subset of the installed datasets. The rationale behind this workflow is shared by similar projects in related fields (e.g. computational linguistics), where data and code are to be strictly separated, allowing researchers to test different approaches and experimental setups with little effort.

### Colexification analysis with CLICS

CLICS is distributed as a standard Python package comprising the pyclics programming library and the clics command-line utility. Both the library and the utility require a CLICS-specific lexical database; the recommended way of creating one is through the load function: calling clics load from the command-line prompt will create a local SQLite database for the package and populate it with data from the installed Lexibank datasets. While this allows researchers with specific needs to select and manually install the datasets they intend, for most use cases we recommend using the curated list of datasets distributed along with the project and found in the clicsthree/datasets.txt file. The list follows the structure of standard requirements.txt files and the entire set can be installed with the standard pip utility.

**Fig. 4:**
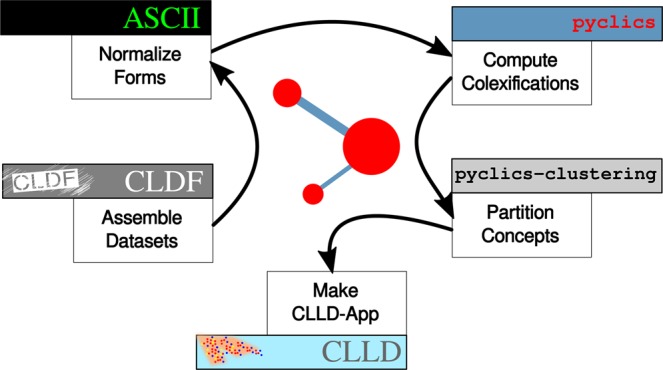
A diagram representing the workflow for installing, preparing, and using CLICS.

The installation of the CLICS tools is the first step in the workflow for conducting colexification analyses. The following points describe the additional steps, and the entire workflow is illustrated in the diagram of Fig. 4.

First, we assemble a set of CLDF datasets into a CLICS database. Once the database has been generated, a colexification graph can be computed. As already described when introducing CLICS^18^ and CLICS2^34^, a colexification graph is an undirected graph in which nodes represent comparable concepts and edges express the colexification weight between the concepts they link: for example, *wood* and *tree*, two concepts that as already mentioned colexify in many languages, will have a high edge weight, while *water* and *dog*, two concepts without a single instance of lexical identity in our data, will have an edge weight of zero.

Second, we normalize all forms in the database. Normalized forms are forms reduced to more basic and comparable versions by additional operations of string processing, removing information such as morpheme boundaries or diacritics, eventually converting the forms from their Unicode characters to the closest ASCII approximation by the unidecode library^59^.

Third, colexifications are then computed by taking the combination of all comparable concepts found in the data and, for each language variety, comparing for equality the cleaned forms that express both concepts (the comparison might involve over two words, as it is common for sources to list synonyms). Information on the colexification for each concept pair is collected both in terms of languages and language families, given that patterns found across different language families are more likely to be a polysemy stemming from human cognition than patterns because of vertical transmission or random resemblance. Cases of horizontal transmission (“borrowings”) might confound the clustering algorithms to be applied in the next stage, but our experience has shown that colexifications are actually a useful tool for identifying candidates of horizontal transmission and areal features. Once the number of matches has been collected, edge weights are adjusted according to user-specified parameters, for which we provide sensible defaults.

The output of running CLICS3 with default parameters, reporting the most common colexifications and their counts for the number of language families, languages, and words, is shown in Table 1.

**Table 1:** The twenty most common colexifications for CLICS3, as the output of command clics colexifications.

Concept A	Concept B	Families	Languages	Words
WOOD	TREE	59	348	361
MOON	MONTH	57	324	327
FINGERNAIL	CLAW	55	236	243
LEG	FOOT	52	349	358
KNIFE (FOR EATING)	KNIFE	51	268	282
SON-IN-LAW (OF MAN)	SON-IN-LAW (OF WOMAN)	49	261	280
SKIN	BARK	49	209	213
WORD	LANGUAGE	49	148	149
ARM	HAND	48	294	300
LISTEN	HEAR	48	107	109
MEAT	FLESH	47	252	262
DAUGHTER-IN-LAW (OF WOMAN)	DAUGHTER-IN-LAW (OF MAN)	47	234	256
SKIN	LEATHER	46	236	258
BLUE	GREEN	46	195	204
MALE (OF ANIMAL)	MALE (OF PERSON)	45	145	163
WOMAN	WIFE	44	289	301
DISH	PLATE	44	155	170
FEMALE (OF PERSON)	FEMALE (OF ANIMAL)	44	146	154
EARTH (SOIL)	LAND	43	159	167
PATH	ROAD	43	133	153

**Fig. 5:**
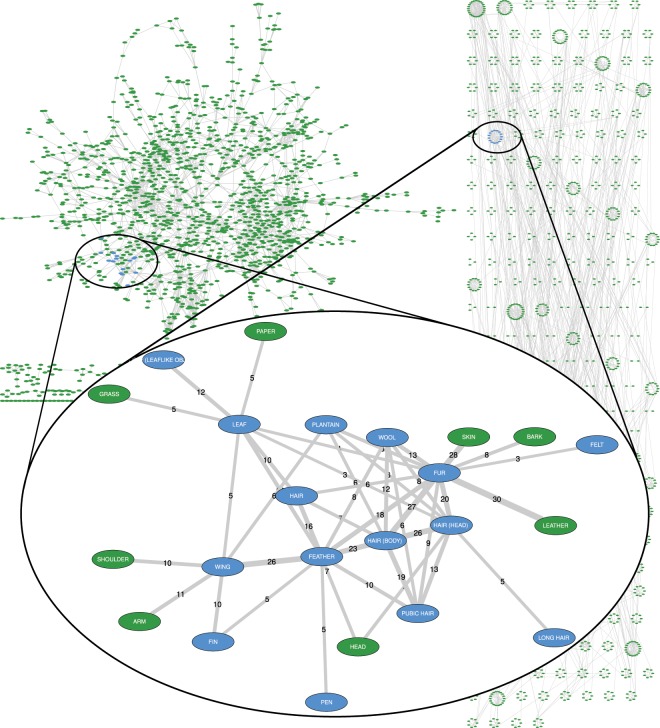
Colexification clusters in CLICS3.

Finally, the graph data generated by the colexification computation, along with the statistics on the score of each colexification and the number of families, languages, and words involved, can be used in different quantitative analyses, e.g. clustering algorithms to partition the graph in “subgraphs” or “communities”. A sample output created with infomap clustering and a family threshold of 3 is illustrated in Fig. 5.

Our experience with CLICS confirms that, as in most real-world networks and particularly in social ones, nodes from colexification studies are not evenly distributed, but concentrate in groups of relatively high density that can be identified by the most adopted methods^60,61^ and even by manual inspection: while some nodes might be part of two or more communities, the clusters detected by the clustering of colexification networks are usually quite distinct one from the other^62,63^. These can be called “semantic communities”, as they tend to be linked in terms of semantic proximity, establishing relationships that, in most cases, linguists have described as acceptable or even expected, with one or more central nodes acting as “centers of gravity” for the cluster: one example is the network already shown in Fig. 1, oriented towards the anatomy of human limbs and centered on the strong *arm-hand* colexification.

CLICS tools provide different clustering methods (see Section *Usage-notes*) that allow to identify clusters for automatic or manual exploration, especially when using its graphical interface. Both methods not only identify the semantic communities but also collect complementary information allowing to give each one an appropriate label related to the semantic centers of the subgraph.

The command-line utility can perform clustering through its cluster command followed by the name of the algorithm to use (a list of the algorithms is provided by the clics cluster list command). For example, clics cluster infomap will cluster the graph with the *infomap* algorithm^64^, in which community structure is detected with random walks (with a community mathematically defined as a group of nodes with more internal than external connecting edges). After clustering, we can obtain additional summary statistics with the clics graph-stats command: for standard CLICS3 with default parameters (and the seed 42 to fix the randomness of the random walk approach) and clustering with the recommended and default *infomap* algorithm, the process results in 1647 nodes, 2960 edges, 92 components, and 249 communities.

The data generated by following the workflow outlined in 4 can be used in multiple different ways (see Section *Usage-notes*), e.g. for preparing a web-based representation of the computed data using the CLLD^65^ toolkit.

## Data Records

CLICS3 is distributed with 30 different datasets, as detailed in Table 2, of which half were added for this new release. Most datasets were originally collected for purpose of language documentation and historical linguistics, such as bodtkhobwa^49^ (derived from^50^), while a few were generated from existing lexical collections, such as logos^66^ (derived from^18^), or from previous linguistic studies, as in the case of wold^67^ (derived from^68^). We selected datasets for inclusion either due to interest for historical linguistics, to maximize the coverage of CLICS2 in terms of linguistic families and areas, or because of on-going collaborations with the authors of the studies.

**Table 2:** Datasets included in CLICS3, along with individual counts for glosses (“Glosses”), concepts mapped to Concepticon (“Concepts”), language varieties (“Varieties”), language varieties mapped to Glottolog (“Glottocodes”), and language families (“Families”); new datasets included for the CLICS3 release are also indicated. Each dataset was published as an independent work on Zenodo, as per the respective citations.

	Dataset	Source	Glosses	Concepticon	Varieties	Glottocodes	Families	New
1	abrahammonpa^84^	^85^	304	304	30	16	2	Yes
2	allenbai^86^	^87^	499	499	9	9	1	
3	bantubvd^88^	^89^	420	415	10	10	1	
4	beidasinitic^41^	^42^	736	735	18	18	1	
5	bodtkhobwa^49^	^50^	553	536	8	8	1	Yes
6	bowernpny^90^	^91^	338	338	175	172	1	
7	castrosui^43^	^44^	510	508	16	3	1	Yes
8	chenhmongmien^92^	^93^	793	793	22	20	1	Yes
9	diacl^45^	^46^	537	537	371	351	25	Yes
10	halenepal^69^	^70^	699	662	13	13	2	Yes
11	hantganbangime^94^	^95^	299	299	22	22	5	Yes
12	hubercolumbian^96^	^97^	346	345	69	65	16	
13	ids^98^	^99^	1310	1308	320	275	60	
14	kraftchadic^100^	^101^	433	428	66	59	2	
15	lexirumah^102^	^103^	604	602	357	231	12	Yes
16	logos^66^	^18^	707	707	5	5	1	Yes
17	marrisonnaga^71^	^72^	580	572	40	39	1	Yes
18	mitterhoferbena^73^	^74^	342	335	13	13	1	Yes
19	naganorgyalrongic^104^	^105^	969	877	10	8	1	Yes
20	northeuralex^56^	^57^	952	951	107	107	21	
21	robinsonap^106^	^107^	391	391	13	13	1	
22	satterthwaitetb^51^	^52^	418	418	18	18	1	
23	sohartmannchin^108^	^109^	279	279	8	7	1	Yes
24	suntb^54^	^55^	929	929	49	49	1	
25	tls^110^	^111^	1140	811	126	107	1	
26	transnewguineaorg^112^	^113^	904	865	1004	760	106	Yes
27	tryonsolomon^114^	^115^	317	314	111	96	5	
28	wold^67^	^68^	1459	1458	41	41	24	
29	yanglalo^47^	^48^	875	869	7	7	1	Yes
30	zgraggenmadang^116^	^117^	311	310	98	98	1	
TOTAL				2906	3156	2271	200	

## Technical Validation

To investigate to which degree our enhanced workflows would improve the efficiency of data creation and curation within the CLDF framework, we conducted two tests. First, we tested the workflow ourselves by actively searching for new datasets which could be added to our framework, noting improvements that could be made for third-party usage and public release. Second, we organized two student tasks with the goal of adding new datasets to CLICS, both involving the delegation of parts of the workflow to students of Linguistics. In the following paragraphs, we will quickly discuss our experiences with these tasks, besides presenting some detailed information on the notable differences between CLICS2 and the improved CLICS3 resulting from both tests.

### Workflow validation

In order to validate the claims of improved reproducibility and the general validity of the workflow for preparing, adding, and analyzing new datasets, we conducted two student tasks in which participants at graduate and undergraduate level were asked to contribute to CLICS3 by using the tools we developed. The first student task was carried out as part of a seminar for doctoral students on *Semantics in Contact*, taught by M. Koptjevskaja-Tamm (MKT) as part of a summer school of the Societas Linguistica Europaea (August 2018, University of Tartu). The second task was carried out as part of an M.A. level course on *Methods in Linguistic Typology*, taught by V. Gast (VG) as a regular seminar at the Friedrich Schiller University (Jena) in the winter semester of 2018/2019.

MKT’s group was first introduced to CLICS2, to the website accompanying the CLICS project, and to the general ideas behind a colexification database. This helped to shape a better understanding of what is curated in the context of CLICS. In a second step, we provided a task description tailored for the students, which was presented by MKT. In a shortened format, it comprised (1) general requirements for CLICS datasets (as described in previous sections), (2) steps for digitizing and preparing data tables (raw input processing), (3) Concepticon linking (aided by semi-automatic mapping), (4) Glottolog linking (identifying languages with Glottocodes), (5) providing bibliographic information with BibTeX, (6) providing provenance information and verbal descriptions of the data.

The students were split into five groups of two people, and each group was tasked with carrying out one of the six tasks for a specific dataset we provided. The students were not given strict deadlines, but we informed them that they would be listed as contributors to the next update of the CLICS2 database if they provided the data up to two months after we introduced the task to them. While the students were working on their respective tasks, we provided additional help by answering specific questions, such as regarding the detailed mapping of certain concepts to Concepticon, via email.

All student groups finished their tasks successfully, with only minor corrections and email interactions from our side. The processed data provided by the students lead to the inclusion of five new datasets to CLICS3: castrosui^43^, a collection of Sui dialects of the Tai-Kadai family spoken in Southern China derived from^44^, halenepal^69^, a large collection of languages from Nepal derived from^70^, marrisonnaga^71^, a collection of Naga languages (a branch of the Sino-Tibetan family) derived from^72^, yanglalo^47^, a dataset of regional varieties of Lalo (a Loloish language cluster spoken in Yunnan, part of the Sino-Tibetan family) derived from^48^, and mitterhoferbena^73^, a collection of Bena dialects spoken in Tanzania derived from^74^.

A similar approach was taken by VG and his group of students, with special emphasis being placed on the difficulties and advantages of a process for collaborative and distributed data preparation. They received instruction material similar to that of MKT’s group, but more nuanced towards the dataset they were asked to work with, namely diacl^45^, a collection of linguistic data from 26 large language families all over the world derived from^46^. Pre-processed data was provided by us and special attention was paid to the process of concept mapping.

In summary, the outcome of the workflow proposed was positive for both groups, and the data produced by the students and their supervisors helped us immensely with extending CLICS3. Some students pointed us to problems in our software pipeline, such as missing documentation on dependencies in our installation instructions. They also indicated difficulties during the process of concept mapping, such as problems arising from insufficient concept definitions for linking elicitation glosses to concept sets. We have addressed most of these problems and hope to obtain more feedback from future users in order to further enhance our workflows.

### CLICS3 validation

**Fig. 6:**
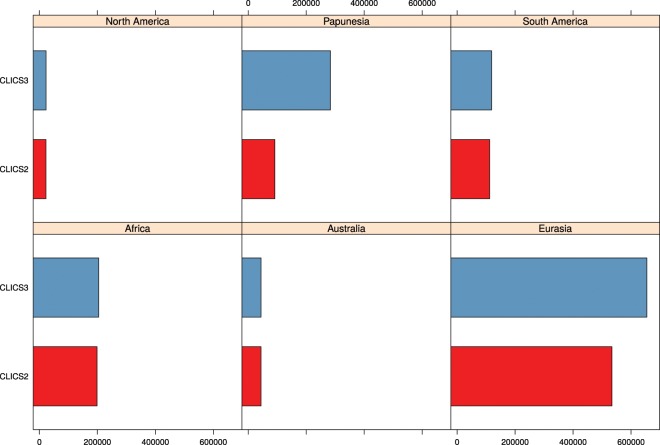
Increase in data points (*values*) for CLICS3.

**Fig. 7:**
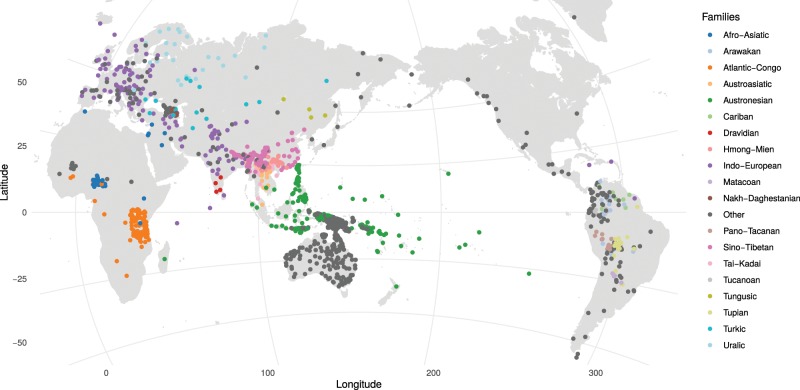
Distribution of language varieties in CLICS3.

The technical validation of CLICS3 is based on functions for deconstructing forms and consequences of this for mapping and finding colexifications. If we compare the data status of CLICS2 with the amount of data available with the release of CLICS3, we can see a substantial increase in data, both regarding the number of languages being covered by CLICS3, and the total number of concepts now included. When looking at the detailed comparisons in Fig. 6, however, we can see that the additions of data occurred in different regions of the world. While we note a major increase of data points in Papunesia, a point of importance for better coverage of “hot spots”^75^, and a moderate increase in Eurasia, the data is unchanged in Africa, North America, and Australia, and has only slightly increased in South America. As can be easily seen from Fig. 7, Africa and North America are still only sparsely covered in CLICS3. Future work should try to target these regions specifically.

While this shows, beyond doubt, that our data aggregation strategy based on transparent workflows that create FAIR data was, by and large, successful, it is important to note that the *average mutual coverage*, which is defined as the average number of concepts for which two languages share a translation^34,76^, is rather low. This, however, is not surprising, given that the original datasets were collected for different purposes. While low or skewed coverage of concepts is not a problem for CLICS, which is still mostly used as a tool for the manual inspection of colexifications, it should be made very clear that quantitative approaches dealing with CLICS2 and CLICS3 need to control explicitly for missing data.

## Usage Notes

The CLICS pipeline produces several artifacts that can serve as an entry point for researchers: a locally browsable interface, well-suited for exploratory research, a SQLite database containing all data points, languoids and additional information, and colexification clusters in the Graph Modelling Language (GML^77^).

The SQLite database can easily be processed with programming languages like R and Python, while the GML representation of CLICS colexification graphs is fully compatible with tools for advanced network analyses, e.g. Cytoscape^78^. Researchers have the choice between different clustering algorithms (currently supported and implemented: highly connected subgraphs^79^, infomap or map equation^64^, Louvain modularity^80^, hierarchical clustering^81^, label propagation^82^, and connected component clustering^83^) and can easily plug-in and experiment with different clustering techniques using a custom package (https://github.com/clics/pyclics-clustering, accessed on November 13, 2019). A sample workflow is also illustrated in the Code Ocean capsule for this publication^37^. For easier accessibility, CLICS data can also be accessed on the web with our CLICS CLLD app, available at https://clics.clld.org/ (accessed on November 15, 2019).

## Data Availability

The workflow by which CLDF datasets are analyzed and published within the CLICS framework, is available as a testable virtual container^36^ that can be freely used on-line in the form of a *Code Ocean capsule*^37^.
